# Missed Massive Morel-Lavallee Lesion

**DOI:** 10.1155/2014/920317

**Published:** 2014-03-30

**Authors:** Shunsuke Takahara, Keisuke Oe, Hironori Fujita, Atsushi Sakurai, Takashi Iwakura, Sang Yang Lee, Takahiro Niikura, Ryosuke Kuroda, Masahiro Kurosaka

**Affiliations:** ^1^Department of Orthopaedic Surgery, Kobe University Graduate School of Medicine, 7-5-1 Kusunoki-cho, Chuo-ku, Kobe 650-0017, Japan; ^2^Department of Orthopaedic Surgery, Hyogo Prefectural Awaji Medical Center, 1-1-137 Shioya, Sumoto 656-0021, Japan

## Abstract

A Morel-Lavallee lesion (MLL) involves posttraumatic fluid collection around the greater trochanter. Many cases of MLL are missed at the initial evaluation, and the treatment of MLL is not well established. We present two cases in which MLL was missed at the initial evaluation. *Case 1.* A 65-year-old man was run over by a parade float. There was subcutaneous hematoma around the left greater trochanter, and no fracture was found. We diagnosed this injury as MLL on the 7th day after the trauma. Although we performed percutaneous drainage, the injured area was infected. *Case 2.* A 57-year-old man was hit by a train in a factory. There was an iliac wing fracture, but an MLL was not initially recognized. On the 6th day after the trauma, when performing open reduction and internal fixation for the iliac fracture, we recognized the lesion and performed percutaneous drainage simultaneously. This lesion also became infected. In these two cases, the wounds finally healed after a long duration of treatment. We suggest that it is important to keep this injury in mind and debride the lesion early and completely in the treatment course.

## 1. Introduction

Closed degloving injury is a severe traumatic separation between the skin and subcutaneous tissue underlying the fascia. Morel-Lavallee lesion (MLL) was first reported by the French physician Maurice Morel-Lavallee in 1853. It is a closed degloving injury occurring over the greater trochanter and is associated with pelvic trauma [1–7]. MLL also occurs around the lower lumbar area [8], calf [9], and knee [10, 11]. MLL typically appears as a fluid collection filled with serous fluid, blood, or necrotic fat [2–4]. Most reports of MLL have been associated with an increased risk of infection [2–15]; therefore, it is important to debride the lesion before it becomes infected.

However, MLL tends to be missed, because the significance of the lesion may not be initially apparent [5]. Additionally, even when the lesion becomes apparent, the treatment of MLL remains controversial.

We present two cases of MLL that required a long duration to heal because of misdiagnosis at the initial evaluation. We also review the literature and discuss the incidence, causes, and treatment of MLL.

## 2. Case Presentation

**Figure 1:**
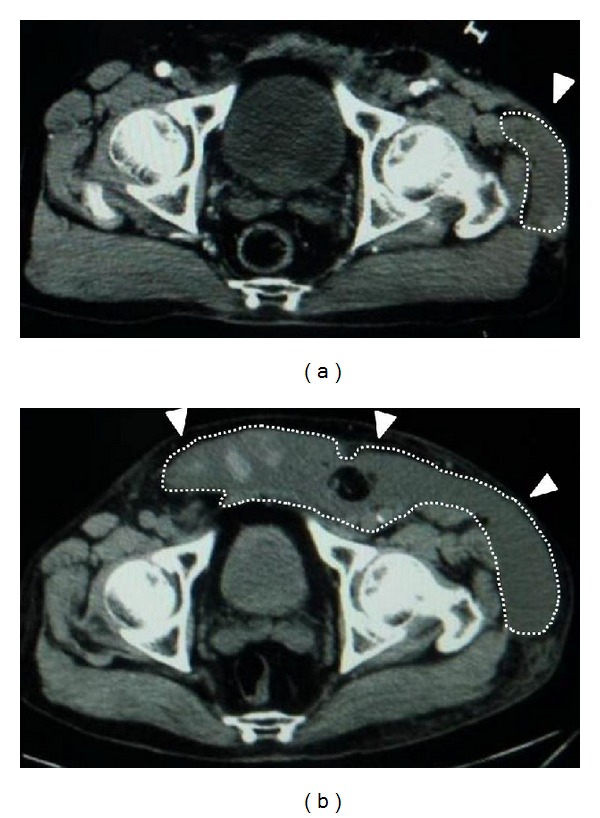
CT axial image around the pelvis. (a) At the initial evaluation, a narrow low-density area was found around the left greater trochanter and no fracture was evident. (b) Seven days after the trauma, the low-density area extended from the lower abdomen to the lateral thigh.

**Figure 2:**
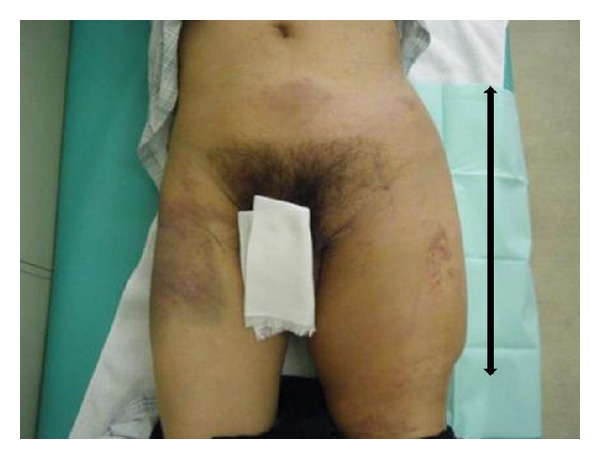
The fluctuant area overlying the lower abdomen to the lateral thigh at 7 days after the trauma.

**Figure 3:**
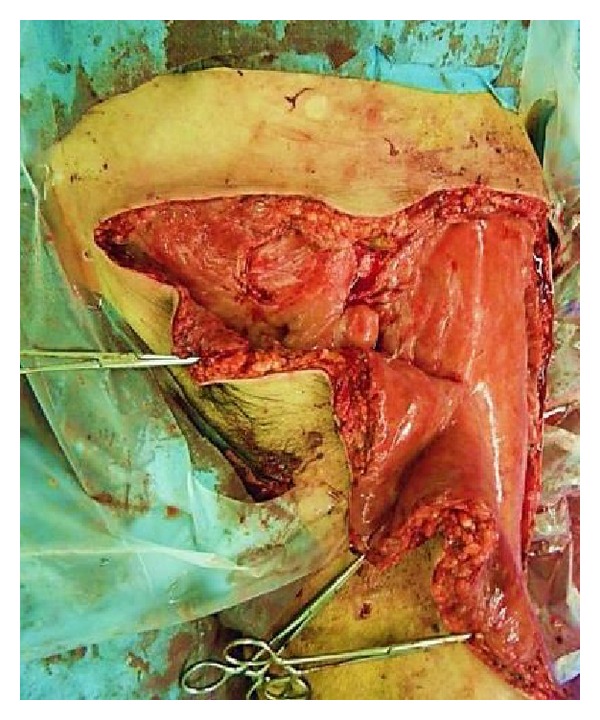
Surgical debridement was performed, and the wound was left open.

**Figure 4:**
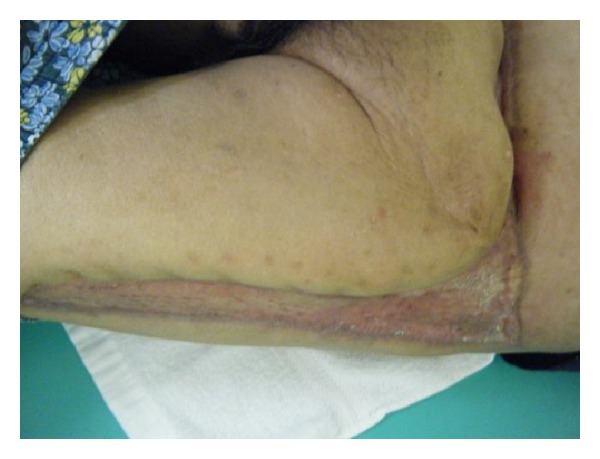
Skin grafting was performed, and the wound healed 4 months after the trauma.

*Case  1.* A 65-year-old man with no underlying disease was run over by a parade float at a festival. Computed tomography (CT) showed a subcutaneous hematoma around the left greater trochanter with no evidence of fracture (Figure 1(a)). The fluctuation in this area gradually spread, and CT examination on the 7th day revealed that the fluid collection extended from the lower abdomen to the lateral thigh (Figure 1(b)) and that the lesion was 40 cm long (Figure 2). Needle aspiration was performed, and 1400 mL of blood was collected from the lesion. The lesion was diagnosed as MLL at this time, and percutaneous drainage was performed through two 2 cm incisions: one over the distal aspect of the lesion and one over the most superior and posterior extent of the lesion after irrigating the interior of the lesion. The drainage was performed for 4 weeks. However, the lesion was infected 4 weeks after the trauma, and methicillin-resistant* Staphylococcus aureus* grew in the culture. We opened the lesion widely and debrided the interior (Figure 3); negative pressure wound therapy was performed thereafter. The infection was well managed, and due to shrinkage of the skin surrounding the lesion, skin grafting was performed 3 months after the trauma. The wound was healed at 4 months after the initial trauma (Figure 4).

**Figure 5:**
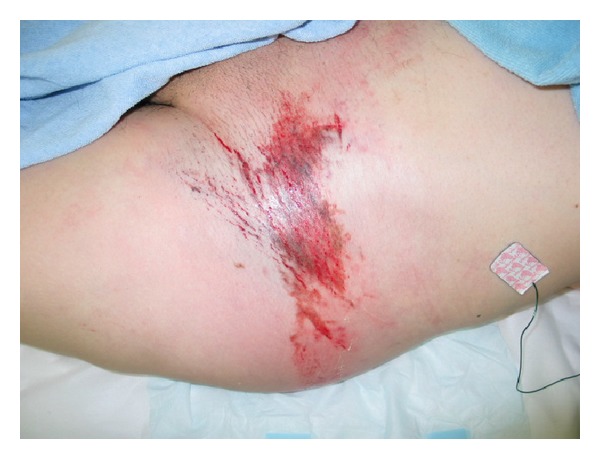
A friction burn was around the left inguinal region.

**Figure 6:**
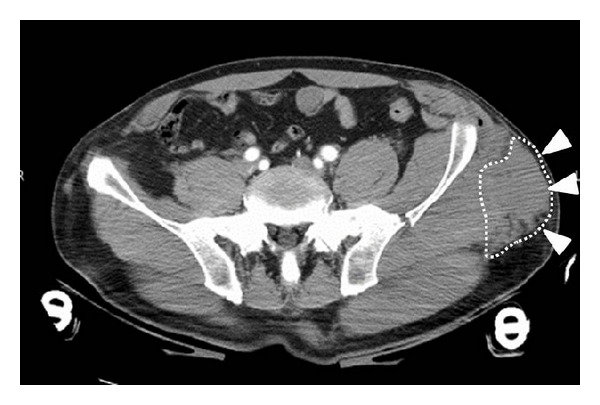
CT axial image around the pelvis. Initial evaluation revealed a narrow low-density area around the left greater trochanter and fracture of the iliac wing.

**Figure 7:**
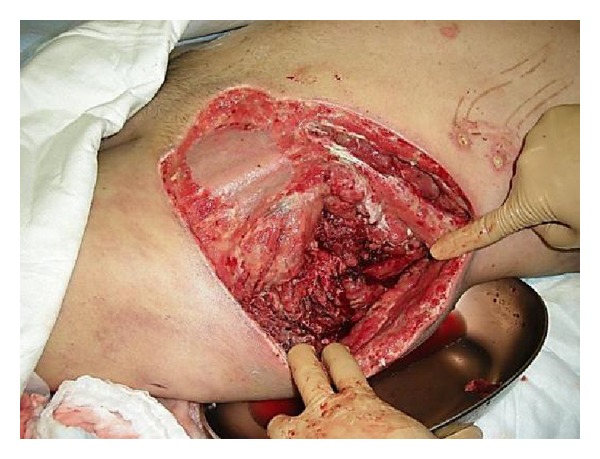
Surgical debridement was performed, and the wound was left open.

**Figure 8:**
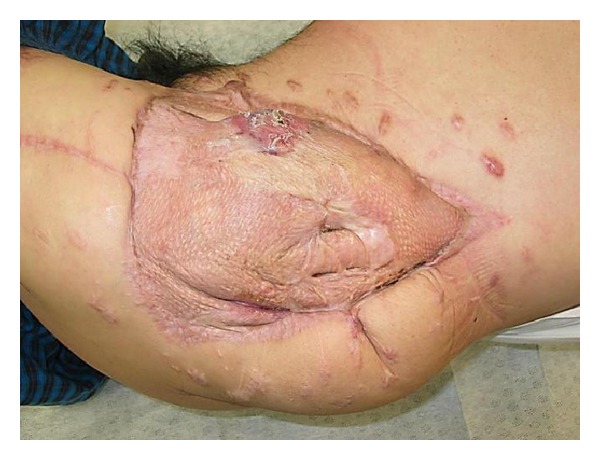
Skin grafting was performed, and the wound healed 4 months after the trauma.

*Case  2*. A 57-year-old man with no underlying disease was hit by a train in a factory. He experienced an iliac wing fracture with a friction burn on the inguinal region (Figure 5). Retrospectively, a subcutaneous hematoma was found around the left iliac wing (Figure 6). As there was no obvious fluctuation, MLL was not initially recognized. Subcutaneous hematoma was revealed around the left greater trochanter when performing open reduction and internal fixation for the iliac fracture on the 6th day after the trauma. We irrigated the interior of the lesion with pulsed lavage and placed a drainage tube through small incisions, similar to Case  1. Because the lesion was infected at 3 days after surgery, we left the lesion exposed (Figure 7). The infection was soon brought under control, and skin grafting was performed at 9 weeks after the trauma. The wound was completely healed at 4 months after the trauma (Figure 8).

## 3. Discussion

Letournel and Judet reported that MLL occurred in 8.3% (23 of 275) of patients who sustained a blow to the trochanter [12]. However, the actual number of lesions is unknown because small-volume lesions are not always detected and recorded [3]. The diagnosis of MLL is usually based on physical examination. Skin mobility, subcutaneous fluctuation, decreased cutaneous sensation, tire marks, and friction burns are useful clinical signs that may help distinguish between closed degloving injuries and contusions [5]. At the initial evaluation, physicians generally treat injuries that are visible on the body surface or on images. In contrast, MLL may be missed because it is not visible on the body surface, and the signs of MLL may not be initially apparent. For example, some patients present with loss of local sensation alone with no external signs of injury [5] or a soft fluctuant area does not appear until several days after the trauma [3]. Kottmeier et al. reviewed 16 MLL cases and reported that the diagnosis of MLL was initially missed in 44% (7 of 16) of the cases [5]. Although MLL is not a rare injury, many MLLs might be missed. In this report, we present two cases in which MLL was not diagnosed for 6 days, because we did not consider the possibility of MLL from the presence of the subcutaneous hematomas. In these two cases, MLL might have been diagnosed earlier if we had considered this injury and performed physical examination repeatedly and more carefully.

MLL is associated with a potential risk of infection [2–15]. Shen et al. reported the presence of infection in 19% of MLL cases (29 of 153) [13]. Suzuki et al. reported that the relative risk of infection is 8 times higher in MLL cases than in non-MLL cases after acetabular fracture fixation [14, 15]. Therefore, it is necessary to diagnose MLL at the early stage of expansion, and surgical treatment should be recommended as early as possible. Tseng and Tornetta reported that cultures obtained from an MLL at the time of debridement (average time from the trauma: 13.1 days) were positive in 46% (11 of 24) of cases [3]. In contrast, Hudson et al. reported that 19% (3 of 16) of wound specimens (debridement was performed within 3 days after the trauma) had a positive culture [2]. Thus, MLL might become gradually contaminated and infected, indicating the necessity for early treatment of the lesion.

Various treatment methods for MLL lesions have been suggested, including aspiration [11], injection of sclerosing agents [7], prolonged closed surgical drainage [2, 4, 5, 7], and open surgical drainage [3, 6, 8]. Several authors have recommended MLL be debrided early after trauma, either before or at the time of fracture fixation [2, 3, 5] and keep the wound open [3, 6, 8]. Other surgeons have recommended early percutaneous drainage with debridement, irrigation, and suction drainage for the treatment of MLL [2, 4, 5, 7]. However, the clinical results are not well established, and the treatment for MLL is controversial. In our two cases, insufficient debridement led to poor results. Open drainage would have a better selection in such massive MLLs with suspected contamination. Regardless of the method used, the necrotic fat and hematoma inside the lesion should be debrided early. Furthermore, to achieve complete debridement, the incision length, number, and location should be determined on a case-by-case basis, and further surgical debridement should be performed if necessary.

In conclusion, our findings suggest that it is important to keep this injury in mind and debride the lesion early and completely in the treatment course.

## References

[1] Morel-Lavallée (1863). Décollements traumatiques de la peau et des couches sous jacentes. *Archives Générales de Médecine*.

[2] Hudson DA, Knottenbelt JD, Krige JEJ (1992). Closed degloving injuries: results following conservative surgery. *Plastic and Reconstructive Surgery*.

[3] Tseng S, Tornetta P (2006). Percutaneous management of Morel-Lavallee lesions. *Journal of Bone and Joint Surgery A*.

[4] Hak DJ, Olson SA, Matta JM (1997). Diagnosis and management of closed internal degloving injuries associated with pelvic and acetabular fractures: the Morel-Lavallee lesion. *Journal of Trauma*.

[5] Kottmeier SA, Wilson SC, Born CT, Hanks GA, Iannacone WM, DeLong WG (1996). Surgical management of soft tissue lesions associated with pelvic ring injury. *Clinical Orthopaedics and Related Research*.

[6] Labler L, Trentz O (2007). The use of vacuum assisted closure (VAC) in soft tissue injuries after high energy pelvic trauma. *Langenbeck’s Archives of Surgery*.

[7] Bansal A, Bhatia N, Singh A, Singh AK (2011). Doxycycline sclerodesis as a treatment option for persistent Morel-Lavallée lesions. *Injury*.

[8] Yilmaz A, Yener O (2013). Giant post-traumatic cyst after motorcycle injury: a case report with review of the pathogenesis. *Prague Medical Report*.

[9] Moriarty JM, Borrero CG, Kavanagh EC (2011). A rare cause of calf swelling: the Morel-Lavallee lesion. *Irish Journal of Medical Science*.

[10] Tejwani SG, Cohen SB, Bradley JP (2007). Management of Morel-Lavallee lesion of the knee: twenty-seven cases in the national football league. *American Journal of Sports Medicine*.

[11] Vanhegan IS, Dala-Ali B, Verhelst L, Mallucci P, Haddad FS (2012). The morel-lavallée lesion as a rare differential diagnosis for recalcitrant bursitis of the knee: case report and literature review. *Case Reports in Orthopedics*.

[12] Letournel E, Judet R (1993). *Fractures of the Acetabulum*.

[13] Shen C, Peng JP, Chen XD (2013). Efficacy of treatment in peri-pelvic Morel-Lavallee lesion: a systematic review of the literature. *Archives of Orthopaedic and Trauma Surgery*.

[14] Suzuki T, Morgan SJ, Smith WR, Stahel PF, Gillani SA, Hak DJ (2010). Postoperative surgical site infection following acetabular fracture fixation. *Injury*.

[15] Suzuki T, Hak DJ, Ziran BH (2009). Outcome and complications of posterior transiliac plating for vertically unstable sacral fractures. *Injury*.

